# The Relation of Big Five Personality Traits on Academic Performance, Well-Being and Home Study Satisfaction in Corona Times

**DOI:** 10.3390/ejihpe14020025

**Published:** 2024-02-15

**Authors:** Johannes Rodrigues, Raffaela Rose, Johannes Hewig

**Affiliations:** Department of Psychology V: Differential Psychology, Personality Psychology and Psychological, Julius-Maximilians University of Würzburg, Pleicherwall 1, 97070 Würzburg, Germany; raffaelarose@yahoo.de (R.R.); hewig@psychologie.uni-wuerzburg.de (J.H.)

**Keywords:** well-being, big five personality, COVID-19, academic performance, home studies

## Abstract

Introduction: As a result of the protective measures taken to contain the COVID-19 pandemic, German students experienced home study in the spring of 2020. The present study addressed the relation between coping with the home study situation and personality. Methods: The interrelations of the Big Five factors with students’ well-being, study satisfaction and academic performance were examined in 287 German online participants. Results: The results showed significant positive correlations of positive affect and conscientiousness, as well as of better academic performance and academic satisfaction. For extraversion, a positive supporting effect on the affective level emerged, although previous studies suggested negative influences of extraversion on affect in home study settings in other phases of the pandemic. Furthermore, in contrast, neuroticism showed a negative relation to study satisfaction and mood in home study. Conclusion: In summary, the personalities of students should be considered in order to provide protective measures and avoid negative coping effects.

## 1. Introduction

The novel COVID-19 virus, which spread rapidly from China in late 2019, developed into a global pandemic in 2020, which was accompanied by enormous restrictions and protective measures. To slow the spread of the so-called “coronavirus”, the population was urged to follow hygiene measures and keep their social contacts to a minimum, which affected almost all areas of life. Gathering in groups was prohibited, curfews were imposed, planned events, both private and public, had to be cancelled, and travel opportunities at home and abroad were restricted [1]. In addition to their private lives, large segments of the population also saw their daily work lives change. For example, the percentage of people working in home offices in Germany more than doubled to 26.1% during the first lockdown compared to the prepandemic period [2]. Yet, while the majority of employed persons in Germany were working regularly at their workplaces despite the pandemic [2], the picture was different among students in Germany. 

At all German universities, teaching had to be unceremoniously converted to online teaching in spring 2020, with a few exceptions, such as lab work. Hence, there could be no more face-to-face teaching, and it came down to the use of online-based formats that were used from home. Study materials were only made available via online platforms and could be accessed and worked through at any time. Libraries, refectories and workrooms remained largely closed, which virtually precluded meeting for learning and exchanging ideas with other students. Although digital teaching was not a completely new concept, as distance learning universities, for example, had been working in this way for some time, the required changeover for students—away from face-to-face study—presented a challenge [3], especially as the sudden need to use this technique was brought about by the pandemic [4,5]. 

### 1.1. Well-Being during COVID-19 Pandemic

Well-being is not singularly definable by a lone metric but encompasses diverse facets that lend themselves to more feasible measurement [6,7]. Depending on the operationalization, different aspects and facets of the concept have been proposed, encompassing hedonistic approaches with a focus on happiness, presence of positive affect and absence of negative affect as well as eudaimonic approaches with focus on life satisfaction [6,8]. In more detail, eudaimonic well-being includes the core dimensions of self-acceptance, purpose in life, autonomy, environmental master, positive relationships and personal growth [9]. Accordingly, following this eudaimonic definition, the World Health Organization (WHO) defined mental health as a state of well-being in which an individual recognizes his or her own abilities, can cope with the normal stresses of life, can work productively and is able to contribute to his or her community. More specifically, the WHO explains that positive mental health can be conceptualized as a subjective sense of well-being. In this vein, Bradburn developed a scale to measure the positive and negative facets of mental well-being [10]. Yet, the hedonistic perspective on well-being still plays a role in science and personal experiences today, and, accordingly, psychological well-being is often recorded using multiple scales including positive and negative affect [11,12,13].

Studies on the psychological well-being of the population during the COVID-19 pandemic showed that the measures of the first lockdown in Germany resulted in the population’s well-being deteriorating significantly and anxiety and depressive symptoms increasing [14,15]. It was further differentiated that lower well-being was due to increased negative affect and concurrently decreased positive affect, which was evident in people’s mood [16]. These developments, as well as increased anxiety scores and rising negative affect among students, were strongly related to restrictions in the university context [17]. Of those studying in Germany, approximately half reported feeling that their workload at university or college had increased significantly since the COVID-19 outbreak. In addition, 54% felt anxious about the expectations in various courses, 47.9% were concerned that they would not be able to successfully complete the academic year, and 47.2% agreed that the change in teaching methods caused them significant stress [14]. The higher-than-average stress and stress experienced due to study conditions during digital teaching was also reflected in decreased student satisfaction [18]. The interrelation between study satisfaction and well-being can be concluded from the work context. Even before the pandemic, it had been demonstrated that job satisfaction and subjective well-being influence each other [11] and job satisfaction is in turn under the influence of working conditions and the work environment [19]. Moreover, psychological well-being and job satisfaction also determine job performance [20,21]. Similarly, for students, lower academic satisfaction is associated with lower academic performance, i.e., lower grades on their examinations [22].

However, against the backdrop of the COVID-19 pandemic, differences in how students cope with the new home study situation and the level of perceived stress have been identified in the past year in Germany [23]. One approach that has been increasingly studied to explain these differences deals with student personality. 

### 1.2. The Influence of the Big Five of Personality during the COVID-19 Pandemic: An Application of Trait Activation Theory

The influences of traits on the emotional reactions, motivational intensity, arousal, attentional scope or behavioral responses of persons have often been shown to also be related to the properties of a situation [24,25,26,27]. Situations may have a specific capability of activating the relevant traits (see trait activation theory, [27]) and, therefore, produce more robust relations of traits and behavior in situations where the specific trait is activated, while in other situations, these relations are not to be found. For example, a person with high trait anger might shout at other people while in a traffic jam and in a hurry, while the shouting is less likely when no time pressure is involved. 

The Big Five model [28] assumes that the individual differences in personalities can be described based on five factors. Two of these factors are of particular interest concerning our study, based on the situation due to the restrictions in social and personal as well as academic life due to the COVID-19 pandemic. 

The factor “conscientiousness“ is a personality dimension that reflects the degree of self-discipline and control. Individuals who are highly conscientious plan events in their lives in advance, are careful, thorough, responsible organized and scrupulous [29]. Furthermore, conscientious individuals exhibit higher levels of diligence, are orderly and behave according to rules and responsibly toward others [30]. The other end of the continuum characterizes people who are irresponsible, disorganized and unscrupulous [29]. Going further, this personality dimension comes into play especially in an occupational context, hence the name “work dimension” in some cases [31]. Conscientiousness is a powerful predictor of work engagement, work performance—independent of performance criteria and job type [32]—and is also considered a conditional personality trait for success in work that is to a considerable extent not performed from the traditional workplace but, for example, from home [33]. Self-management is an indispensable criterion for the successful completion of such tasks and is attributed to individuals with a high degree of conscientiousness [34]. Concerning compliance with pandemic restrictions, conscientious people are expected to have fewer problems with restrictions due to the pronounced conformity to rules and their sense of responsibility [35]. Furthermore, conscientiousness helps to withstand the negative psychological influence of the COVID-19 pandemic [36]. Hence, conscientiousness was a protective factor against the “general” situation brought about by the COVID-19 pandemic. Yet, other traits were expected to relate more differentially to the specific phase of the restrictions that were placed on social life due to COVID-19.

“Extraversion” measures an individual’s sociability. Extraverts are very energetic, optimistic, friendly, assertive and sociable [37]. In contrast, so-called introverts, individuals with a low expression of this factor, tend to be reserved, socially independent and fluctuate in their pace of work, although they are not sluggish [31,37]. In relation to the limitations imposed by the pandemic on social interaction and social support, extraverts could be more heavily influenced by the pandemic as they rely on social support in stressful situations [38], and the perceived accessibility to such social support is responsible for the extent to which extraverts can cope with negative stressful situations [39]. Accordingly, in the context of the initial lockdown of the 2020 emerging pandemic, extraverts experienced higher stress levels [40,41,42]. Hence, a dampened response concerning well-being and performance was expected.

Other aspects of the Big Five personality traits that are not directly the focus of this work, as we deemed the situational trait activation not as pronounced, are openness, agreeableness and neuroticism. The openness to experience factor includes traits such as intellectual curiosity, divergent thinking, a willingness to consider new ideas and a strong imagination. Individuals high on this factor think unconventionally and independently, whereas individuals low on openness to experience are more conventional and prefer the familiar [31,37]. Agreeableness is associated with positive social interaction, i.e., trusting, helpful, soft-hearted and sympathetic people; conversely, a low level represents distrustful, hostile, skeptical, uncooperative and unhelpful people [31,37]. Neuroticism forms a measure of an individual’s emotional stability and personal adjustment, which is why this dimension is sometimes referred to as “emotional stability”. High levels of neuroticism are reflected in strong mood swings and characterize people who are very volatile in their emotions. In contrast, individuals with low neuroticism are calm, well-adjusted individuals who are not prone to extreme and inappropriate emotional states [31,37].

### 1.3. Personality and Home Study: An Application of Trait Activation Theory

Regarding students during the pandemic, who mostly studied from home, extraversion and conscientiousness came into focus concerning trait activation in this context because of the explained characteristics of the individual personality dimensions of the Big Five. Due to the lack of infrastructure at the universities, socializing with other students was curbed during pandemic online semesters [23], leading to a high trait-activation situation for this personality trait. Students with high extraversion consequently felt more stressed by the pandemic than introverted fellow students [43], and during home study well-being was negatively related to extraversion [44]. Students additionally expressed motivational or attentional problems with home office learning during the COVID-19 pandemic [43], possibly due to the lack of external control of their learning activities by their teachers. This situation provides a high trait-activation characteristic for conscientiousness. However, even before the Corona crisis, conscientiousness was found to be related to motivation and satisfaction in online learning and performance [45,46]. Yet, the motivational self-regulation and self-efficacy of more conscientious students likewise favored better handling of the home study situation in the context of pandemic online semesters [47].

To conclude with respect to the previously mentioned literature, we were especially interested in the influence of extraversion and conscientiousness on home study success, focusing, however, not only on the well-being aspect but also on the performance. 

### 1.4. Hypotheses

Given the previous findings, we expected a relationship between extraversion and negative psychological well-being as well as a relationship between conscientiousness and positive psychological well-being [44]. Further, it was expected that differences in student satisfaction in the home study situation could also be revealed based on students’ personality traits. Considering the results of previous studies and the influence of well-being on satisfaction [11], similar patterns as those for well-being were expected. Given the influence of students’ satisfaction with their study situation on their academic performance [22], an effect was also expected in this context for the personality traits extraversion and conscientiousness like the two previous hypotheses with conscientiousness leading to better performance, while extraversion should lead to worse performance. A summary of the hypotheses is shown in Table 1.

Other traits were not expected to have a specific influence on academic performance, well-being or satisfaction in the home study context, as we deemed the trait activation of the situation not specific and strong enough for them. However, with this decision we had neglected the influence of neuroticism, which was addressed exploratorily and is addressed in the Limitations section.

## 2. Materials and Methods

### 2.1. Ethical Statement

The study was carried out in accordance with the recommendations of the ethical guidelines of the psychological association of the country of the institution, with written informed consent from all subjects. All subjects gave written informed consent in accordance with the Declaration of Helsinki before they participated in the experiment. The protocol was approved by the local ethics committee (GZEK-2021-32 on the 20.05.2021).

### 2.2. Preregistration

The preregistration (blinded) of the study is given here: https://osf.io/fbsdp/?view_only=e564fd3d22ba471fb767c5256a39b9a8 (accessed on 12 February 2024).

### 2.3. Participants

A-priory sampling size calculation with *r* = 0.17 [41,44], α = 0.05 and a power of 0.80 led to 269 participants using G*Power [48] (https://osf.io/fbsdp/?view_only=e564fd3d22ba471fb767c5256a39b9a8, accessed on 12 February 2024). 

A total of 287 complete datasets were collected (220 female, 65 male, 2 no information; age mean = 22.679 years; age SD = 3.47; age range = 18–48 years; for further details, see Supplemental Material S1). The results for academic performance, however, were based on 260 records collected because of missing data. In particular, we excluded data (17 participants) for that specific scale that could not be interpreted due to not following the given grade format, i.e., data that fell outside the outlier limits of the grading scale. Participants were students at German universities that had experienced at least one complete online semester during the pandemic. They could receive participation credits, but no monetary compensation was offered. In order to characterize the sample collected, their age, gender, course of study, the semester they were currently in and the federal state of their university were collected. Furthermore, the predominant living situation of the students during the online semesters in the pandemic was also asked, whereby the participants could choose between “shared apartment”, “living alone” and “in the parental home” or “living with the family” or “other living situation”. The demographical data can be seen in Supplement S1.

### 2.4. Materials

#### 2.4.1. Questionnaires/Items

The descriptive statistics and internal consistencies (reliabilities: Cronbach’s α and McDonald’s ω) of the questionnaire data are shown in Table S2 in Supplemental Materials.

#### 2.4.2. Big Five Personality Factors

The Big Five Inventory-SOEP (BFI-S, [49]) was used to assess the personality dimensions of the Big Five. The short scale measures the personality factors neuroticism (example item: I am someone who often worries), extraversion (example item: I am someone who is communicative and talkative), openness (example item: I am someone who is original, brings in new ideas), conscientiousness (example item: I am someone who completes tasks effectively and efficiently), and agreeableness (example item: I am someone who is considerate and kind to others) based on 15 items. For each trait, respondents indicate on the BFI-S the extent to which they attribute the described trait to themselves on a seven-point scale ranging from “1 = not at all true” to “7 = strongly true”. Each personality factor is addressed by three items with different polarities.

#### 2.4.3. Well-Being

Students’ subjective well-being was assessed using two survey instruments. The Current Mood Scale (ASTS, [50]) was used, which captures the current mood, i.e., the state portion of the respondents’ subjective well-being using a descriptive word for the current mood. The self-report measure comprises 19 items, which are assigned to five subscales: sadness (TR, example item: saddened), hopelessness (HO, example item: discouraged), fatigue (MT, example item: exhausted), anger (ZO, example item: angry) and positive mood (PO, example item: cheerful). The 19 items are adjectives that reflect emotional states and are to be rated on a seven-point rating scale from “1 = not at all” to “7 = very strongly”. The individual dimensions can be considered separately but can also be used to form an overall measure to describe current negative mood (NE). 

The second instrument used to assess students’ well-being was the German version of the Positive and Negative Affect Schedule (PANAS, [51]). The PANAS questionnaire consists of 20 items that capture both positive (example item: joyfully excited) and negative affect (example item: irritated) independently. For this purpose, adjectives describing different sensations and feelings are queried and assessed using a 5-level response format for a specific period of time—in this case during the home study of the last semesters. Ten adjectives each capture the dimensions of Positive Affect (PA) and Negative Affect (NA). The response alternatives are “1 = not at all”, “2 = a little”, “3 = to some extent”, “4 = considerably” and “5 = extremely”. 

#### 2.4.4. Study Satisfaction

Two measurement instruments were also used to assess student satisfaction. First, the general study satisfaction was determined with the help of the Short Questionnaire for the Assessment of Study Satisfaction (FB-SZ-K, [52]), which consists of 9 items, each of which includes three statements on satisfaction with the study content (example item: I find my studies really interesting), the study conditions (example item: My university pays too little attention to the needs of students) and coping with the study load (example item: I often feel tired and exhausted from studying). The items are to be answered by indicating on a scale from “0 = the statement does not apply at all” to “10 = the statement applies completely” to what extent the respective statement sentences correspond to the opinion of the students. 

As a second measurement instrument for study satisfaction, the Job Satisfaction Questionnaire [53] was used in a modified form. The original questionnaire is based on the seven subscales “my colleagues”, “my supervisor”, “my job”, “my working conditions”, “organization and management”, “my development” and “my pay”, for which an answer must be selected on a four-point Likert scale between the choices “1 = yes”, “2 = rather yes”, “3 = rather no” and “4 = no”. At the end of each scale, a summary satisfaction question is asked, represented by Kunin faces. The answers here range from “1 = lowest satisfaction” to “7 = highest satisfaction” but without the verbal labeling [53]. For the present research question, the scales “my job”, “my working conditions” and “organization and management” were singled out and adapted to the research question about home study satisfaction. For this purpose, “my job” was used to inquire about the content of the home study, “my working conditions” was used to inquire about the home office, and “organization and management” was used to inquire about the university’s handling of the pandemic or the online semester. The exact reformulations of the questionnaire items used can be found in Supplemental Materials S3. Furthermore, instead of a 7-point scale, a 5-point scale (“1 = lowest satisfaction” to “5 = highest satisfaction”) was used for the nonverbal Kunin faces of the summary satisfaction questions. 

#### 2.4.5. Academic Performance

In the context of this study, academic performance was referred to as examination performance, i.e., the grades achieved by the students. The grades were scored from 1 (best) to 6 (worst). The respondents were asked in advance to provide the grades they had achieved in the previous semester, the winter semester 2020/2021, and, thus, the second semester that had taken place in the home study so that first-year students were not included in the survey. In the survey of grades, all university achievements of the winter semester were to be entered as far as possible. A maximum of seven entry fields were available for this purpose, and at least one had to be completed. The grades were to be entered with one decimal place, which is why students should, if possible, only refer to numerically recorded performances and leave out performances that were recorded as “passed” or “not passed”. If the students did not have any of the grades just described in the previous semester, they had the option of indicating this in the first input field. For each grade given, it was also asked whether the subject in which the grade was achieved corresponded to the student’s personal interest. Students were asked to select whether their interest in the subject was “rather high”, “average” or “rather low”. Finally, for each grade entered, the students were asked to indicate how representative the grade was of their own performance assessment in this subject, i.e., whether the grade was “in line” with their own assessment, “better than expected” or “worse than expected”. A mean score was then calculated from the indicated grades, which allowed for subsequent comparison of academic performance, with a lower grade point average representing better academic performance than a higher grade point average. 

### 2.5. Procedure

Participants were recruited via personal contacts as well as social media and circulars at German universities. They were students who, at the time of the survey, had already studied at a university or college in the previous semester, the winter semester 2020/21, and had thus experienced at least one complete online semester during the pandemic. Students could receive participation credits, but no monetary compensation was offered. 

To collect the data, an online questionnaire was compiled using SoSci Survey [54], which could be completed via the internet platform *soscisurvey.de.* The processing time for the questionnaire took about 30 min. 

### 2.6. Statistical Analysis and Evaluation

The study was preregistered on the Open Science Framework (OSF) prior to the survey at https://osf.io/fbsdp/?view_only=e564fd3d22ba471fb767c5256a39b9a8, (accessed on 12 February 2024). Associations between variables were tested using Pearson correlation coefficient *r*. A significance level of α = 0.05 was set for all analyses, with Bonferroni–Holm post hoc alpha correction applied for multiple testing (see Supplemental Table S4). Data processing, as well as statistical analysis, were performed entirely using *Jamovi* [55]. The Results Section 3.1. Confirmatory Analysis deals with the results of the preregistered hypotheses.

Exploratory Pearson correlations of the five personality scales of the BFI-S with the individual scales of the ASTS were also analyzed, and relationships between the constructs of well-being, study satisfaction and academic performance were examined. In addition, the relationship between the personality factors conscientiousness and extraversion with aspects of well-being not considered in the hypotheses above (preregistration: 1a and 1b) was considered. The result Section 3.2. Exploratory Analysis deals with these analyses.

## 3. Results

### 3.1. Confirmatory Analysis 

**Figure 1:**
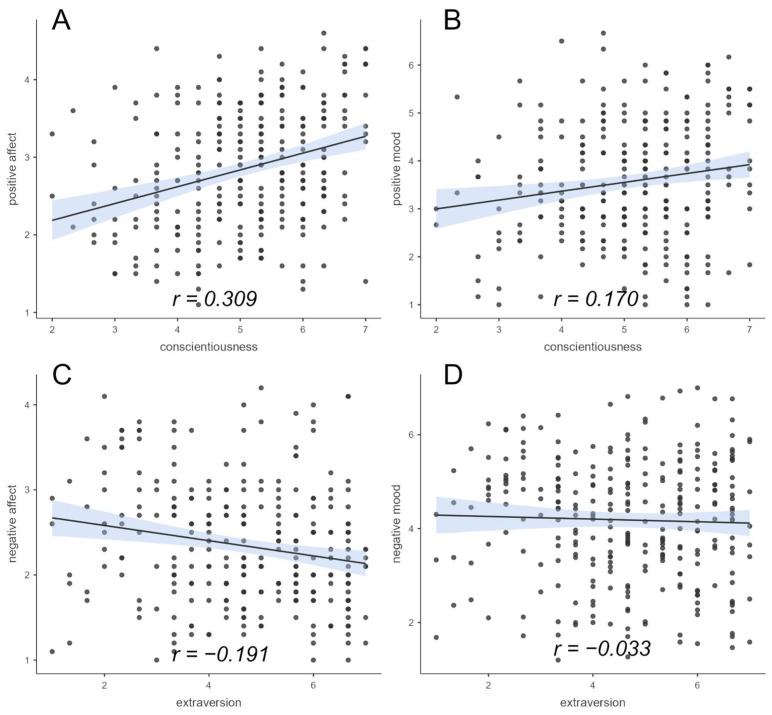
Correlations between conscientiousness and positive well-being and extraversion and negative well-being (positive/negative affect and positive/negative mood). (**A**): Correlation of conscientiousness and positive affect, (**B**): Correlation of conscientiousness and positive mood, (**C**): Correlation of extraversion and negative affect, (**D**): Correlation of extraversion and negative mood. The blue shaded area represents the respective standard error.

The first hypothesis included the assumption that the personality factor conscientiousness is related to a higher sense of well-being. The correlation between conscientiousness and positive affect was significantly positive (*r =* 0.309, *p_holm_* < 0.001), as was the correlation between conscientiousness and positive mood (*r* = 0.170, *p_holm_* < 0.05); see Figure 1.

Regarding well-being and extraversion, lower well-being was expected, and, thus, a positive relationship between extraversion and negative affect and between extraversion and negative mood was hypothesized. Contrary to the hypothesis, no positive relationship between extraversion and negative affect (*r* = −0.191, *p_holm_* = 0.999) was found (see Figure 1). The correlation between extraversion and negative mood (see Figure 1) was also not positively correlated (*r* = −0.063, *p_holm_* = 1). 

**Figure 2:**
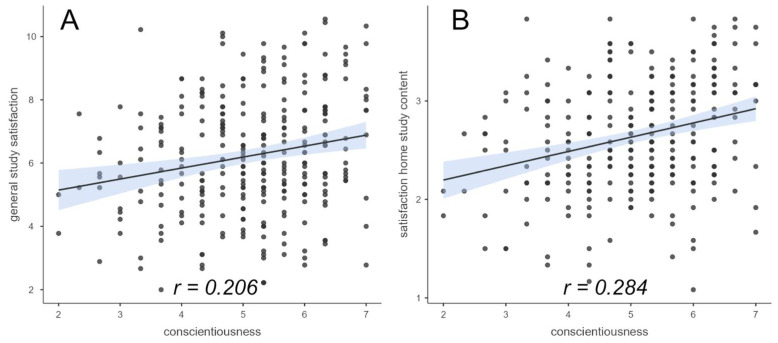
Correlations between conscientiousness and general study satisfaction, as well as home study content. (**A**): Correlation of conscientiousness and general study satisfaction, (**B**): Correlation of conscientiousness and satisfaction with home study content. The blue shaded area represents the respective standard error.

The second hypothesis dealt with study satisfaction as a function of the personality traits conscientiousness and extraversion. Study satisfaction was assessed on the one hand by means of general study satisfaction, and on the other hand individual aspects of the home study were surveyed. As expected, the results showed a positive correlation between conscientiousness and general study satisfaction (*r* = 0.206, *p_holm_* < 0.01) during the online semesters in home study (see Figure 2). The satisfaction with one aspect of home study, home study content (*r* = 0.284, *p_holm_* < 0.001), also correlated significantly positively with conscientiousness (see Figure 2). However, although being in the correct direction, after alpha correction, the two other aspects, namely university (*r* = 0.147, *p_holm_* = 0.058) and home office (*r* = 0.114, *p_holm_* = 0.212), were no longer significant (for details, see Table S4).

Regarding the personality factor extraversion, a negative correlation was suspected with Home Study satisfaction, yet no significant effect was found (*r* = 0.055, *p_holm_* = 1). Moreover, no significant correlation could be found for home study content (*r* = −0.023, *p_holm_* = 1), university (*r =* 0.038, *p_holm_* = 1) and home office (*r =* −0.064, *p_holm_* = 1).

**Figure 3:**
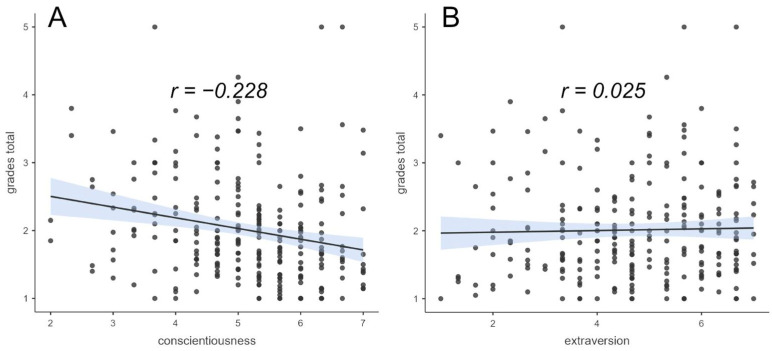
Correlations between conscientiousness and extraversion and academic performance (grade point average of performance achieved; lower grade indicates better academic performance). (**A**): Correlation of conscientiousness and grade points average of performance achieved, (**B**): Correlation of extraversion and grade points average of performance achieved. The blue shaded area represents the respective standard error.

The results for academic performance were based on 260 records collected because of missing data. In particular, we excluded data that could not be interpreted due to not following the given grade format, i.e., data that fell outside the outlier limits of the grading scale. The empirical range of the academic performance was from 1 (best) to 5 (mean = 2.012, SD = 0.762). We expected better academic performance in students in a home study situation with high conscientiousness and poorer performance with high extraversion. Since in Germany the lower the grade point average, the better the performance, a negative relationship with grade point average was hypothesized for conscientiousness and a positive relationship was hypothesized for extraversion. The results (see Figure 3) showed, as expected, a significant negative correlation between grade point average and conscientiousness (*r* = −0.228, *p_holm_* < 0.01), yet no positive correlation with extraversion was found (*r* = 0.025, *p_holm_* = 1). 

Lastly, it was hypothesized that there would be no significant relationship between the personality traits of agreeableness, openness and neuroticism and the constructs of well-being, study satisfaction and academic performance in the home study setting. Therefore, using positive and negative affect for well-being, overall study satisfaction for academic satisfaction and grade point average for academic performance, a correlational analysis was conducted including 90% confidence intervals (90% CI) and no alpha correction. This procedure was used as the hypothesis was that no correlation existed; hence, a test on H0 was performed. 

However, only for the personality factor openness was positive affect unrelated (*r* = 0.093, *p_uncorrected_* = 0.114, 90% CI [−0.004, 0.189]), as expected. For all other relations, significant results were found (see Table 2).

### 3.2. Exploratory Analysis 

In the exploratory examination of affect in relation to conscientiousness and extraversion without underlying hypotheses, there was a significant positive correlation between extraversion and positive affect (*r* = 0.205, *p_uncorrected_* < 0.001) as well as a negative correlation with negative affect (*r* = −0.191, *p_uncorrected_* = 0.001) and a negative correlation between conscientiousness and negative affect (*r* = −0.175, *p_uncorrected_* = 0.003).

Furthermore, the correlations between the individual scales of the ASTS and the five personality factors were examined. Regarding conscientiousness, significant negative correlations were found with the subscales sadness (*r* = −0.183, *p_uncorrected_* = 0.002), hopelessness (*r* = −0.173, *p_uncorrected_* = 0.003), fatigue (*r* = −0.121, *p_uncorrected_* = 0.04) and the total scale for negative mood (*r* = −0.163, *p_uncorrected_* = 0.006); the personality factor extraversion did not correlate significantly with any individual scale of the ASTS. For the Big Five factor agreeableness, there was only a significant negative correlation with the anger subscale (*r* = −0.234, *p_uncorrected_* < 0.001). When considering the personality expression of openness, significant positive correlations were shown with the sadness (*r* = 0.154, *p_uncorrected_* = 0.009), hopelessness (*r* = 0.137, *p_uncorrected_* = 0.020) and fatigue (*r* = 0.144, *p_uncorrected_* = 0.014) subscales, as well as the negative mood total scale (*r* = 0.124, *p_uncorrected_* = 0.035). The strongest correlations were found in the exploratory analysis of the subscales of the ASTS and the relationship with neuroticism. Neuroticism was significantly and positively correlated with both sadness (*r* = 0.406, *p_uncorrected_* < 0.001) and hopelessness (*r* = 0.367, *p_uncorrected_* < 0.001) as well as fatigue (*r* = 0.364, *p_uncorrected_* < 0.001), yet only a weakly significant positive correlation was found for anger (*r* = 0.167, *p_uncorrected_* = 0.005). Regarding the total negative mood scale, a significant positive correlation was found with neuroticism (*r* = 0.378, *p_uncorrected_* < 0.001) and with the positive mood scale, neuroticism correlated significantly negatively (*r* = −0.274, *p_uncorrected_* < 0.001).

The correlations between the constructs of well-being, study satisfaction and academic performance were also analyzed independently of personality. Here, general study satisfaction correlated strongly and significantly with positive and negative aspects of well-being, both for affect (PA: *r* = 0.478, *p_uncorrected_* < 0.001; NA: *r* = −0.513, *p_uncorrected_* < 0.001) and mood (PO: *r* = 0.504, *p_uncorrected_* < 0.001; NE: *r* = −0.668, *p_uncorrected_* < 0.001). For correlations with academic performance, no significant correlations were shown with well-being other than for hopelessness leading to worse performance (*r*(259) = 0.142, *p_uncorrected_* = 0.022); however, overall academic satisfaction was slightly significantly correlated with a better grade point average (*r*(259) = −0.154, *p* = 0.013). 

For further details, see Table S5 in Supplemental Materials.

## 4. Discussion

The aim of the work was to show the differential relations of the personality of the students on dealing with the home study situation that arose during the COVID-19 pandemic in Germany. The focus was on the relation of the personality factors extraversion and conscientiousness with well-being, study satisfaction and academic performance. 

The results showed that students with high conscientiousness scores were better able to cope with the challenges of home study. Conscientiousness was conducive to well-being, study satisfaction and better academic performance in such a situation. Regarding extraversion, the results suggested that well-being was increased by extraversion, and the expected negative effect was not observed. 

### 4.1. Personality and Well-Being in Home Study

The results indicate that positive psychological well-being during the pandemic home study situation was strongly related to the personality factor conscientiousness in students, thus, confirming our hypothesis. The results fit with the findings that pandemic adversity did not affect the well-being of conscientious individuals [36]. One possible explanation for this effect is rule compliance and sense of responsibility, which are associated with high levels of the personality factor [30]. As complying with the restrictions does not prove to be a burden for conscientious individuals, psychological well-being did not suffer from the home study situation and conscientious students might not have found it difficult to cope with the imposed changes [44]. In contrast, it has been suggested that students who are highly sociable and usually derive their support in difficult situations from social interactions, i.e., are highly extraverted, suffered from the restrictions during online semesters, especially in the social domain, and this manifests itself in negative psychological well-being. Contrary to these assumptions [43,44] this study did not show a positive relationship between extraversion and negative well-being. On the contrary, the personality factor extraversion revealed a similar supporting pattern regarding well-being as already observed for conscientiousness, suggesting that higher extraversion expression is associated with higher positive well-being and low negative well-being. Although the effect is not as pronounced as for conscientiousness, it nevertheless suggests that extraversion is beneficial to well-being during home study, rather than being a hindrance as expected. Comparable studies have explained the findings in terms of the positive influence on psychological well-being fundamentally attributed to extraversion, which was attenuated by the constraints and stresses of the pandemic but still protectively present [16]. Yet, the exploratory positive association found between positive affect and extraversion can also be explained by the phase of the pandemic in which the study was conducted. After all, it was already the third semester that took place under pandemic conditions. Extraverts may have found ways to cope with the situation and maintain social contacts also using digital infrastructure. In addition, the progress of the vaccination and the decreasing number of infections gave reason to hope that normality would soon return, which may have strengthened the positive affect. Although the focus of this paper was on the personality factors extraversion and conscientiousness, the salient associations of neuroticism with almost every construct examined are worth noting. That neuroticism is strongly related to well-being was not explicitly expected in the context of this work but was also not surprising given the emotional instability that characterizes this personality factor. Regardless of a pandemic and its associated negative consequences, neuroticism is considered an important predictor of subjective well-being, with pronounced neuroticism being associated with negative affect [12]. Therefore, it is quite conceivable that students with higher neuroticism scores would suffer more from the constraints and uncertainty of the pandemic or the home study situation and exhibit more negative well-being [56]. 

### 4.2. Personality and Study Satisfaction in Home Study

Higher conscientiousness was associated with greater study satisfaction in home study. This pattern was evident in both overall satisfaction and satisfaction with individual aspects of study, content, home office and university, yet partly being not that pronounced. Satisfaction with the type of study tasks and their content, on the other hand, clearly correlated with students’ conscientiousness. Because home study was also partly asynchronous, self-discipline and organization, partial characteristics of the conscientiousness factor, also played a major role in the completion of the study tasks during the pandemic [57]. However, the expectation of a negative relationship between extraversion and study satisfaction in home study could not be confirmed by the present study. Extraversion was not related to study satisfaction during home study at all. This was shown both in very low correlations between extraversion and the queried satisfactions regarding studying in the past semesters and is in line with recent findings [58]. They also fit with the higher well-being for extraversion and consequently do not lead to a negative relation with study satisfaction [11]. Furthermore, and in line with the underlying relationship between well-being and satisfaction [58], we found that the personality factor neuroticism also showed a strong negative correlation with study satisfaction in home study. This may further indicate that the risk factor of neuroticism would lead to generally unfavorable feelings in this rather uncertain situation.

### 4.3. Personality and Academic Performance in Home Study

Regarding academic performance, the study was able to confirm, as hypothesized, a clear relationship between conscientiousness and better grades in home study. On the one hand, this can be explained by the relationship between study satisfaction and better academic performance [22], which was also exploratively found in this current study. Since the results also showed that highly conscientious students did not experience tremendous negativity in well-being or dissatisfaction with home study, the role of conscientiousness in academic performance during online semesters is additionally important. Yet, conscientiousness is also considered to be the Big Five personality factor predictive of successful academic performance, independent of a pandemic or general home study situation [59], so the finding is just as expected. Yet, the effect seems to be significantly dampened compared to normal circumstances (*r*_Kappe & Van Der Flier 2012_ = 0.47, *r*_study_ = 0.228, *z* = 2.609, *p* = 0.005). Concerning extraversion, we did not find the expected tendency that high extraversion expression was associated with a lower grade point average in home study. Thus, the results are not in line with the predicted relation between extraversion and poorer academic performance in the online conditions during the pandemic [60]. The reason for this lack of finding could be manifold. One reason could be that the proposed lack of performance could be due to the higher effort extraverts had to invest in their social stimulation at the beginning of the pandemic times. This may have deprived them of academic success early, but as the social networks were established, adapted and in use in the ongoing pandemic situation, many coping mechanisms might have been at hand. For example, learning groups that were cancelled at the beginning of the pandemic were restarted at later stages of the pandemic in smaller online groups. Hence, this missing motivation that may have caused differences in performance was regained at later stages of the pandemic. Hence, datasets that were recorded at earlier times in the pandemic might be more suited to explore the question about the negative influence or relations of extraversion [60].

### 4.4. Practical Implications

While the primary focus of this study has centered on specific aspects and correlations concerning personality and home study during the pandemic, the findings offer insightful contributions to both the behavior during the pandemic and the broader field of home study itself. One can deduct additional rules to the recommendation of adaptability, diverse learning modalities, flexible staffing and learning models, interactive virtual learning experiences, addressing inequalities, general support, simplification and standardization as well as quality assurance [5]. As demonstrated in this study, neuroticism emerges as a potentially influential personality factor in coping with the pandemic and home study situation. This could lead to the possibility of higher support for students with this personality trait in order to help overcome learning difficulties and problems arising from new learning formats, such as digital inverted classrooms and other learning formats not focusing on personal presence. This support is especially important if personal or global crises are to be expected or experienced. Hence, teachers may want to consider the personality of their students more in their teaching approach to ensure that risk factors do not lead to an unfavorable outcome. In addition, protective factors, such as conscientiousness and extraversion (for mood enhancing), may also be identified to allow students with these feature a less controlled and/or supported learning experience to enhance their sense of agency in learning. Finally, implementing and reinforcing learning groups combining students of different personality traits may help to let all students benefit from their differences in personality in their learning experiences. Yet, this is only possible if the individual person characteristics are considered. Therefore, this important aspect has to be implemented in teaching concepts.

### 4.5. Limitations and Criticism

One limitation is that the relation with academic performance was not investigated with the expected 269 but with only 260 participants. Missing data led to an insufficient sample size concerning academic performance, although only 9 persons are missing. Nevertheless, we were not able to pursue our data collection due to time constraints, as we did not want to extend our data collection to an additional semester, especially in those pandemic times, where drastic changes occurred in the rules that applied as well as the perception of the rules. Additionally, there is a heavy gender bias in the study. This may have had an influence on the results, especially for the three personality factors extraversion, agreeableness and neuroticism [61,62].

Furthermore, the validity and comparability of academic performance should also be questioned in that the number of grades reported as well as the subjects, their difficulty and the type of examination performance varied between students, potentially constituting a confounding variable. Online semesters included changing many examination formats, reverting to online-based performance reviews or written assignments, which could have an impact on the grades achieved regardless of Home Study.

Also, an important aspect in the limitation of this work is that the role of personality was only examined correlatively, and, thus, no causal conclusions or directions of action can be drawn between the constructs. Although it is reasonable to assume that personality is an underlying aspect that persists over time, such a causal relationship was not tested in the analysis of the data of this study and, therefore, cannot be interpreted. Further, although the presumed correlations could be partially confirmed, a different underlying reason could be given. For example, in addition to personality, events in private life may also be related to well-being and study satisfaction and influence the experience of the home study situation in this respect.

Since the study was a snapshot and there were no repeated measurements at different times before and during the pandemic, the sole influence of the pandemic home study cannot be assumed beyond doubt. Moreover, the different phases of the pandemic, which also varied in the severity and extent of their restrictions, may have been perceived differently, which makes the findings not generally indicative of home study in general or of the period of the pandemic but only of the home study during the period and phase of the survey. This period, in turn, extended into the summer months, during which the restrictions were less severe, the weather allowed for meeting outside, and the vaccination progress allowed students more and more freedom. Moreover, we are not able to separate the effect of the pandemic clearly from general home study effects. Hence, this confounding factor is observed in our data and poses a great limitation.

With respect to the methodological approaches of the study, it can be assumed that the multiple assessments of a construct such as well-being, using the ASTS, the PANAS and the associated Bonferroni–Holm alpha correction for multiple testing, resulted in too strict an adjustment and that interesting significant results were overlooked. Yet, for that reason, the exploratory correlational results were included to provide a better overall view of the data. Future studies could additionally include repeated measures of well-being, study satisfaction and academic performance to provide a wider view of the home study situation and performance linked to personality. In addition, a more detailed survey of personality traits with a more comprehensive questionnaire than the BFI-S could grant more precise assessments and more quality, whereas the survey of well-being with only one instead of two survey instruments should be sufficient. However, such studies would have to deal with the problem of a very long-lasting longitudinal design.

## 5. Conclusions

Although this work focused on specific aspects and correlations related to personality and home study during the pandemic, the results provide interesting insights into the field of home study per se. Yet, the present study only provides a starting point for many interesting questions. For example, a more detailed analysis of possible factors influencing the living situation on the subjective home study experience could be made, or the remaining three Big Five factors—agreeableness, openness, and neuroticism—could be more closely examined in the context of home study or home study during the pandemic. As has been shown in the present study, neuroticism, for example, could possibly be one of the most influential personality factors in coping with the pandemic and the home study situation. Despite our global efforts to overcome the COVID-19 pandemic, some teaching formats are likely to not return to face-to-face after the pandemic, as a major boost to digitization in the university context has been provided by this situation. It is, therefore, reasonable to assume that home study and asynchronous learning and teaching will continue to be a part of student life in the future. The examination of home study, therefore, holds great potential to ensure equality concerning performance opportunities for different personality types, for example, by providing help in the form of guidance for persons needing it. The relevance of this study becomes even more apparent when considered against the backdrop of the ongoing theoretical controversy surrounding home study. The transition to digital platforms and the continued prevalence of home study post-pandemic present a paradigm shift in the educational landscape. As this shift unfolds, the theoretical frameworks that underpin home study need to be revisited and refined. This study, by addressing the nuanced interplay between personality traits and home study outcomes, offers a crucial contribution to the ongoing theoretical debate.

To conclude, differences in how students deal with the home study situation in pandemic times were revealed with respect to the big five personality traits. The personality factor conscientiousness emerged as a beneficial factor on multiple levels, while extraversion might act as a mood enhancing trait. Neuroticism, however, emerged as a risk factor for performance and mood on multiple levels in home study. Not all students will be able to cope equally well with changes and university challenges in the future, and individual consideration, guidance and personalized design of the learning environment, therefore, may be beneficial when designing study conditions or the home study environment. Such revelations contribute substantively to the theoretical discourse, particularly in understanding the nuanced relationship between personality factors and the evolving landscape of home study.

## Figures and Tables

**Table 1:** Summary of hypotheses.

Trait	Hypotheses
**conscientiousness**	H1.1: The higher the trait conscientiousness, the higher the well-being.
	H1.2: The higher the trait conscientiousness, the higher the satisfaction with home study.
	H1.3: The higher the trait conscientiousness, the better the academic performance in the past online semester.
**extraversion**	H2.1: The higher trait extraversion, the lower well-being.
	H2.2: The higher the trait extraversion, the lower the satisfaction with home study.
	H2.3: The higher the trait extraversion, the worse the academic performance in the previous online semester.
**Openness** **neuroticism** **agreeableness**	H3: No significant correlation between well-being, satisfaction with studies and academic performance and the personality traits openness, neuroticism and agreeableness is expected due to the home study situation.

**Table 2:** Correlation patterns and 90% confidence intervals of well-being and general study satisfaction with agreeableness, neuroticism and openness.

		Agreeableness	Openness	Neuroticism
**positive affect**	Pearson’s r	0.138 *	0.093	−0.317 ***
	*p*-value	0.020	0.114	0.000
	90% CI upper	0.232	0.189	−0.227
	90% CI lower	0.041	−0.004	−0.402
**negative affect**	Pearson’s r	−0.154 **	0.136^*^	0.522 ***
	*p*-value	0.009	0.021	0.000
	90% CI upper	−0.057	0.23	0.59
	90% CI lower	−0.247	0.039	0.448
**general study satisfaction**	Pearson’s r	0.123 *	−0.126^*^	−0.388 ***
	*p*-value	0.038	0.033	0.000
	90% CI upper	0.217	−0.029	−0.302
	90% CI lower	0.026	−0.221	−0.467

*Note.* * *p* < 0.05, ** *p* < 0.01, *** *p* < 0.001.

## Data Availability

The data, materials and the statistical analyses are available on the OSF repository (non-frozen version of the project: https://osf.io/zektw/, accessed on 12 February 2024). For further interest beyond these analyses and data, contact the first author.

## Supplementary Materials

The following supporting information can be downloaded at https://www.mdpi.com/article/10.3390/ejihpe14020025/s1, Supplemental Information S1: Demographical information in detail; Table S2: Reliability of the questionnaires; Supplemental Information S3: Detailed information about the questionnaires; Table S4: Bonferroni–Holm correlation adjustment procedure for extraversion and conscientiousness; Table S5: Explorative correlation analysis without alpha adjustment.

## References

[1] Usher K., Bhullar N., Jackson D. (2020). Life in the Pandemic: Social Isolation and Mental Health. J. Clin. Nurs..

[2] Möhring K., Naumann E., Reifenscheid M., Blom A.G., Wenz A., Rettig T., Lehrer R., Krieger U., Juhl S., Friedel S. (2020). Die Mannheimer Corona-Studie: Schwerpunktbericht Zu Erwerbstätigkeit und Kinderbetreuung [The Mannheim Corona Study: Report of Occupation and Child Care].

[3] Sherry L. (1995). Issues in Distance Learning. Int. J. Educ. Telecommun..

[4] Cramarenco R.E., Burcă-Voicu M.I., Dabija D.-C. (2023). Student Perceptions of Online Education and Digital Technologies during the COVID-19 Pandemic: A Systematic Review. Electronics.

[5] Tang K.H.D. (2023). Impacts of COVID-19 on Primary, Secondary and Tertiary Education: A Comprehensive Review and Recommendations for Educational Practices. Educ. Res. Policy Pract..

[6] Deci E.L., Ryan R.M. (2008). Hedonia, Eudaimonia, and Well-Being: An Introduction. J. Happiness Stud..

[7] Diener E. (1984). Subjective Well-Being. Psychol. Bull..

[8] Waterman A.S. (1993). Two Conceptions of Happiness: Contrasts of Personal Expressiveness (Eudaimonia) and Hedonic Enjoyment. J. Personal. Soc. Psychol..

[9] Ryff C.D., Singer B.H. (2008). Know Thyself and Become What You Are: A Eudaimonic Approach to Psychological Well-Being. J. Happiness Stud..

[10] World Health Organization (2004). Promoting Mental Health: Concepts, Emerging Evidence, Practice (Summary Report).

[11] Bowling N.A., Eschleman K.J., Wang Q. (2010). A Meta-Analytic Examination of the Relationship between Job Satisfaction and Subjective Well-Being. J. Occup. Organ. Psychol..

[12] Librán E.C. (2006). Personality Dimensions and Subjective Well-Being. Span. J. Psychol..

[13] Carter N.T., Guan L., Maples J.L., Williamson R.L., Miller J.D. (2016). The Downsides of Extreme Conscientiousness for Psychological Well-Being: The Role of Obsessive Compulsive Tendencies. J. Personal..

[14] Matos Fialho P.M., Spatafora F., Kühne L., Busse H., Helmer S.M., Zeeb H., Stock C., Wendt C., Pischke C.R. (2021). Perceptions of Study Conditions and Depressive Symptoms During the COVID-19 Pandemic Among University Students in Germany: Results of the International COVID-19 Student Well-Being Study. Front. Public Health.

[15] Schwinger M., Trautner M., Kärchner H., Otterpohl N. (2020). Psychological Impact of Corona Lockdown in Germany: Changes in Need Satisfaction, Well-Being, Anxiety, and Depression. Int. J. Environ. Res. Public Health.

[16] Anglim J., Horwood S. (2021). Effect of the COVID-19 Pandemic and Big Five Personality on Subjective and Psychological Well-Being. Soc. Psychol. Personal. Sci..

[17] Li H.Y., Cao H., Leung D.Y.P., Mak Y.W. (2020). The Psychological Impacts of a COVID-19 Outbreak on College Students in China: A Longitudinal Study. Int. J. Environ. Res. Public Health.

[18] Loton D., Parker P., Stein C., Gauci S. (2020). Remote Learning during COVID-19: Student Satisfaction and Performance. EdArXiv.

[19] Aziri B. (2011). Job Satisfaction, A Literature Review. Manag. Res. Pract..

[20] Judge T.A., Thoresen C.J., Bono J.E., Patton G.K. (2001). The Job Satisfaction–Job Performance Relationship: A Qualitative and Quantitative Review. Psychol. Bull..

[21] Wright T.A., Cropanzano R. (2000). Psychological Well-Being and Job Satisfaction as Predictors of Job Performance. J. Occup. Health Psychol..

[22] Martirosyan N.M., Saxon D., Wanjohi R. (2014). Student Satisfaction and Academic Performance in Armenian Higher Education. Am. Int. J. Contemp. Res..

[23] Traus A., Höffken K., Thomas S., Mangold K., Schröer W. (2020). Stu.diCo—Studieren Digital in Zeiten von Corona [Digital Studies in Times of Corona].

[24] Coan J.A., Allen J.J.B., McKnight P.E. (2006). A Capability Model of Individual Differences in Frontal EEG Asymmetry. Biol. Psychol..

[25] Mussel P., Reiter A.M.F., Osinsky R., Hewig J. (2015). State- and Trait-Greed, Its Impact on Risky Decision-Making and Underlying Neural Mechanisms. Soc. Neurosci..

[26] Rodrigues J., Allen J.J.B., Müller M., Hewig J. (2021). Methods Matter: An Examination of Factors That Moderate Predictions of the Capability Model Concerning the Relationship of Frontal Asymmetry to Trait Measures. Biol. Psychol..

[27] Tett R.P., Guterman H.A. (2000). Situation Trait Relevance, Trait Expression, and Cross-Situational Consistency: Testing a Principle of Trait Activation. J. Res. Personal..

[28] Costa P.T., McCrae R.R. (1992). Revised NEO Personality Inventory (NEO-PI-R) and NEO Five-Factor Inventory (NEO-FFI).

[29] Roccas S., Sagiv L., Schwartz S.H., Knafo A. (2002). The Big Five Personality Factors and Personal Values. Personal. Soc. Psychol. Bull..

[30] Roberts B.W., Jackson J.J., Fayard J.V., Edmonds G., Meints J. (2009). Conscientiousness. Handbook of Individual Differences in Social Behavior.

[31] Tett R.P., Burnett D.D. (2003). A Personality Trait-Based Interactionist Model of Job Performance. J. Appl. Psychol..

[32] Barrick M.R., Mount M.K., Judge T.A. (2001). Personality and Performance at the Beginning of the New Millennium: What Do We Know and Where Do We Go Next?. Int. J. Sel. Assess..

[33] Clark L., Karau S., Michalisin M.D. (2012). Telecommuting Attitudes and the “Big Five” Personality Dimensions. J. Manag. Policy Pract..

[34] Lamond D., Daniels K., Lamond D., Standen P. (2000). Personality and Telework. Managing Telework: Perspectives from Human Resource Management and Work Psychology.

[35] Krupić D., Žuro B., Krupić D. (2021). Big Five Traits, Approach-Avoidance Motivation, Concerns and Adherence with COVID-19 Prevention Guidelines during the Peak of Pandemic in Croatia. Personal. Individ. Differ..

[36] Zhang X., Wang Y., Lyu H., Zhang Y., Liu Y., Luo J. (2021). The Influence of COVID-19 on the Well-Being of People: Big Data Methods for Capturing the Well-Being of Working Adults and Protective Factors Nationwide. Front. Psychol..

[37] McCrae R.R., John O.P. (1992). An Introduction to the Five-Factor Model and Its Applications. J. Personal..

[38] Amirkhan J.H., Risinger R.T., Swickert R.J. (1995). Extraversion: A “Hidden” Personality Factor in Coping?. J. Personal..

[39] Swickert R.J., Rosentreter C.J., Hittner J.B., Mushrush J.E. (2002). Extraversion, Social Support Processes, and Stress. Personal. Individ. Differ..

[40] Liu S., Lithopoulos A., Zhang C.Q., Garcia-Barrera M.A., Rhodes R.E. (2021). Personality and Perceived Stress during COVID-19 Pandemic: Testing the Mediating Role of Perceived Threat and Efficacy. Personal. Individ. Differ..

[41] Weiß M., Rodrigues J., Hewig J. (2022). Big Five Personality Factors in Relation to Coping with Contact Restrictions during the COVID-19 Pandemic: A Small Sample Study. Soc. Sci..

[42] Zacher H., Rudolph C.W. (2021). Big Five Traits as Predictors of Perceived Stressfulness of the COVID-19 Pandemic. Personal. Individ. Differ..

[43] Fichter L., Zeichhardt R., von Bernstorff C. (2021). Studieren Oder Isolieren? Persönlichkeits-Effekte Beim Erleben Der Pandemie. [Study or Isolation? Effects of Personality on Perception and Experience of the Pandemic]. Die Neue Hochsch..

[44] Gupta K., Parimal B.S. (2020). Relationship between Personality Dimensions and Psychological Well-Being among University Students during Pandemic Lockdown. J. Glob. Resour..

[45] Schniederjans M.J., Kim E.B. (2005). Relationship of Student Undergraduate Achievement and Personality Characteristics in a Total Web-Based Environment: An Empirical Study. Decis. Sci. J. Innov. Educ..

[46] Shih H.-F., Chen S.-H.E., Chen S.-C., Wey S.-C. (2013). The Relationship among Tertiary Level EFL Students’ Personality, Online Learning Motivation and Online Learning Satisfaction. Procedia-Soc. Behav. Sci..

[47] Staller N., Großmann N., Eckes A., Wilde M., Müller F.H., Randler C. (2021). Academic Self-Regulation, Chronotype and Personality in University Students During the Remote Learning Phase Due to COVID-19. Front. Educ..

[48] Faul F., Erdfelder E., Buchner A., Lang A.-G. (2009). Statistical Power Analyses Using G*Power 3.1: Tests for Correlation and Regression Analyses. Behav. Res. Methods.

[49] Schupp J., Gerlitz J.Y. (2014). Big Five Inventory-SOEP (BFI-S).

[50] Dalbert C. (2002). ASTS—Aktuelle Stimmungsskala [Present Mood Scales].

[51] Breyer B., Bluemke M. (2016). Deutsche Version Der Positive and Negative Affect Schudule PANAS (GESIS Panel).

[52] Westermann R., Heise E., Spies K. (2018). FB-SZ-K—Kurzfragebogen Zur Erfassung Der Studienzufriedenheit [Short Scales to Assess Study Satisfaction].

[53] Neuberger O., Allerbeck M. (2014). Arbeitszufriedenheit [Work Satisfaction].

[54] Leiner D.J. (2021). SoSci Survey.

[55] The Jamovi Project (2021). Jamovi, Version 1.8. https://www.jamovi.org.

[56] Modersitzki N., Phan L.V., Kuper N., Rauthmann J.F. (2021). Who Is Impacted? Personality Predicts Individual Differences in Psychological Consequences of the COVID-19 Pandemic in Germany. Soc. Psychol. Personal. Sci..

[57] Audet É.C., Levine S.L., Metin E., Koestner S., Barcan S. (2021). Zooming Their Way through University: Which Big 5 Traits Facilitated Students’ Adjustment to Online Courses during the COVID-19 Pandemic. Personal. Individ. Differ..

[58] Sahinidis A.G., Tsaknis P.A. (2021). Exploring the Relationship of the Big Five Personality Traits with Student Satisfaction with Synchronous Online Academic Learning: The Case of COVID-19-Induced Changes. Strategic Innovative Marketing and Tourism in the COVID-19 Era.

[59] Kappe R., Van Der Flier H. (2012). Predicting Academic Success in Higher Education: What’s More Important than Being Smart?. Eur. J. Psychol. Educ..

[60] Yu Z. (2021). The Effects of Gender, Educational Level, and Personality on Online Learning Outcomes during the COVID-19 Pandemic. Int. J. Educ. Technol. High. Educ..

[61] Vianello M., Schnabel K., Sriram N., Nosek B. (2013). Gender Differences in Implicit and Explicit Personality Traits. Personal. Individ. Differ..

[62] Weisberg Y., DeYoung C., Hirsh J. (2011). Gender Differences in Personality across the Ten Aspects of the Big Five. Front. Psychol..

