# Supplementary material for: The Relation of Big Five Personality Traits on Academic Performance, Well-Being and Home Study Satisfaction in Corona Times
Source: Eur J Investig Health Psychol Educ. 2024 Feb 15;14(2):368–84. doi: 10.3390/ejihpe14020025 (PMC10887932; doi:10.3390/ejihpe14020025)
Supplement: Supplementary file 1 [file ejihpe-14-00025-s001.zip › ejihpe-2799734-supplementary.pdf]

S1: Demographical information in detail.

The vast majority of the sample studied in Bavaria (71.1%), and a wide variety of study programs was indicated. In looking at the living situation, 97 (33.8%) of the participants lived predominantly in a shared apartment during the home study, 61 (21.3%) lived alone, and 104 (36.2%) reported living with family or in their parents' home during the online semesters. The remaining 25 individuals (8.7%) selected "other living situation."

Table S1A: Frequencies of Bundesland.

Levels	Counts	% of Total	Cumulative %
Baden-Württemberg	9	3.1 %	3.1 %
Bayern	204	71.1 %	74.2 %
Berlin	2	0.7 %	74.9 %
Brandenburg	1	0.3 %	75.3 %
Hamburg	1	0.3 %	75.6 %
Hessen	4	1.4 %	77.0 %
Nordrhein-Westfalen	61	21.3 %	98.3 %
Rheinland Pfalz	1	0.3 %	98.6 %
Sachsen	2	0.7 %	99.3 %
Thüringen	1	0.3 %	99.7 %
Wien	1	0.3 %	100.0 %

Table S1B: Frequencies of living situation.

Levels	Counts	% of Total	Cumulative %
shared apartment	97	33.8 %	33.8 %
alone	61	21.3 %	55.1 %
with family	104	36.2 %	91.3 %
others	25	8.7 %	100.0 %

Table S2: Reliability of the questionnaires

Reliabilities (Cronbach's α; McDonald's ω), means (*M*) and standard deviations (*SD)* for the Big Five factors (extraversion, conscientiousness, neuroticism, openness, agreeableness), positive

and negative affect, positive and negative mood, overall study satisfaction and satisfaction with home study content, home office and university.

**Personality trait**	***Cronbach´s α***	***McDonald´s ω***	***M***	***SD***
Extraversion (BFI-S)	.878	.878	4.66	1.51
Conscientiousness (BFI-S)	.696	.705	5.10	1.11
Neuroticism (BFI-S)	.774	.780	4.54	1.32
Openness (BFI-S)	.630	.647	4.84	1.13
Agreeableness (BFI-S)	.611	.649	5.39	.987
Positive affect (PANAS)	.904	.905	2.86	.776
Negative affect (PANAS)	.841	.842	2.34	.707
Positive mood (ASTS)	.920	.923	3.57	1.20
Negative mood (ASTS)	.892	.896	4.39	1.33
Sadness (ASTS)	.917	.919	4.24	1.69
Hopelessness (ASTS)	.861	.861	3.95	1.69
Fatigue (ASTS)	.915	.918	4.95	1.48
Anger (ASTS)	.905	.906	3.34	1.64
General study satisfaction (FB-SZ-K)	.642	.647	6.22	1.87
Satisfaction Content (see S3)	.876	.878	2.65	.565
Home office satisfaction (see S3)	.835	.839	2.95	.545
Satisfaction university (see S3)	.933	.933	2.53	.653

Materials S3: Detailed information about the questionnaires

Demographical data

In order to characterize the sample collected, their age, gender, course of study, the semester they were currently in, and the federal state of their university were collected. Furthermore, the predominant living situation of the students during the online semesters in the pandemic was also asked, whereby the participants could choose between "shared apartment", "living alone" and "in the parental home" or "living with the family" or "other living situation". The demographical data can be seen in supplement S1.

Big Five personality factors

The Big Five Inventory-SOEP (BFI-S; Schupp & Gerlitz, 2014) was used to assess the personality dimensions of the Big Five. The short scale measures the personality factors neuroticism, extraversion, openness, conscientiousness and agreeableness, based on 15 items. For each trait, respondents indicate on the BFI-S the extent to which they attribute the described trait to themselves on a seven-point scale ranging from "1 = not at all true" to "7 = strongly true". Each personality factor is addressed by three items with different polarities.

Well-being

Students' subjective well-being was assessed using two survey instruments. The Current Mood Scale (ASTS; Dalbert, 2002) was used, which captures the current mood, i.e., the state portion of the

respondents' subjective well-being. The self-report measure comprises 19 items, and the overall measures are assigned to five subscales: sadness (TR), hopelessness (HO), fatigue (MT), anger (ZO), and positive mood (PO). The 19 items are adjectives that reflect emotional states and are to be rated on a seven-point rating scale from "1 = not at all" to "7 = very strongly".

The second instrument used to assess students' well-being was the German version of the Positive and Negative Affect Schedule (PANAS; Breyer & Bluemke, 2016). The PANAS questionnaire consists of 20 items that capture both positive and negative affect independently. For this purpose, adjectives describing different sensations and feelings are queried and assessed using a five-level response format for a specific period of time—in this case, during the home study of the last semesters. Ten adjectives each capture the dimensions of Positive Affect (PA) and Negative Affect (NA). The response alternatives are "1 = not at all", "2 = a little", "3 = to some extent", "4 = considerably" and "5 = extremely".

Study satisfaction

Two measurement instruments were also used to assess student satisfaction. First, the general study satisfaction was determined with the help of the Short Questionnaire for the Assessment of Study Satisfaction (FB-SZ-K; Westermann et al., 2018), which consists of nine items, each of which includes three statements on satisfaction with the study content, the study conditions and coping with the study load. The items are to be answered by indicating on a scale from "0 = the statement does not apply at all" to "10 = the statement applies completely" to what extent the respective statement sentences correspond to the opinion of the students.

As a second measurement instrument for study satisfaction, the Job Satisfaction Questionnaire (Neuberger & Allerbeck, 2014) was used in a modified form. The original questionnaire is based on the seven subscales "my colleagues", "my supervisor", "my job", "my working conditions", "organization and management", "my development" and "my pay", for which an answer must be selected on a four-point Likert scale between the choices "1 = yes", "2 = rather yes", "3 = rather no" and "4 = no". At the end of each scale, a summary satisfaction question is asked, represented by Kunin faces. The answers here range from "1 = lowest satisfaction" to "7 = highest satisfaction" but without the verbal labeling (Neuberger & Allerbeck, 2014). For the present research question, the scales "my job", "my working conditions" and "organization and management" were singled out and adapted to the research question about home study satisfaction. For this purpose, "my job" was used to inquire about the content of the home study, "my working conditions" was used to inquire about the home office, and "organization and management" was used to inquire about the university's handling of the pandemic or the online semester. The exact reformulations of the questionnaire items used can be seen below at the end of this material. Furthermore, instead of a 7-point scale, a 5-point scale ("1 = lowest satisfaction" to "5 = highest satisfaction") was used for the nonverbal Kunin faces of the summary satisfaction questions.

Academic achievement

In the context of this study, academic performance was referred to as examination performance, i.e., the grades achieved by the students. The respondents were asked in advance to provide the grades they had achieved in the previous semester, the winter semester 2020/2021, and, thus, the second semester that had taken place in the home study so that first-year students were not included in the survey. In the survey of grades, all university achievements of the winter semester were to be entered as far as possible. A maximum of seven entry fields were available for this purpose, and at least one had to be completed. The grades were to be entered with one decimal place, which is why students should, if possible, only refer to numerically recorded performances and leave out performances that were recorded as "passed" or "not passed". If the students did not have any of the grades just described in the previous semester, they had the option of indicating this in the first input field. For each grade given, it was also asked whether the subject in which the grade was achieved corresponded to the student's personal interest. Students were asked to select whether their interest in the subject was "rather high", "average" or "rather low". Finally, for each grade entered, the students were asked to indicate how representative the grade was of their own performance assessment in this subject, i.e., whether the grade was "in line" with their own assessment, "better than expected" or

"worse than expected". A mean score was then calculated from the indicated grades, which allowed for subsequent comparison of academic performance, with a lower grade point average representing better academic performance than a higher grade point average.

Reformulated questionnaire on job satisfaction

My home study activity: This refers to the content of your home study, the nature of your study tasks.

- I like
- Boring
- Stuck
- Independently
- Useless
- Viewed at
- Disappointing
- Underchallenges me
- See results
- Can use my skills
- Can realize my ideas
- Responsible

All in all, how satisfied are you with your job?

My home office: This refers to the conditions under which you study at home (e.g. aids/equipment, workspace, environment, noise, temperature, etc.).

- Comfortable
- Bad
- Clean
- Pleasant
- Troubled
- Turbulent
- A lot of noise
- Plenty of room
- Hazardous to health
- Pleasant temperature
- Tiring

All in all, how satisfied are you with your home office conditions?

University: This means how you see the university as a whole, how the cooperation between the departments and faculties works during the online semesters, how you assess regulations, planning and information and the "top" management during the online semesters.

- Weak
- Interested in the opinion of students

- Insufficient information
- Progressive
- Circumstantial
- Poor teaching climate
- In confusion
- Pride in the university
- I feel good here
- We can have a say
- Poor planning
- Does little for the students
- Powerful

All in all, how satisfied are you with your university's handling of online semesters?

References

Breyer, B., & Bluemke, M. (2016). Deutsche Version der Positive and Negative Affect Schudule PANAS (GESIS Panel). *Zusammenstellung Sozialwissenschaftlicher Items Und Skalen*, *October*, 1–16. https://doi.org/10.6102/zis242

Dalbert, C. (2002). *ASTS - Aktuelle Stimmungsskala [present mood scales]*. Leibniz-Zentrum für Psychologische Information und Dokumentation (ZPID) (Hrsg.), Elektroni-sches Testarchiv. Trier: ZPID. https://doi.org/10.23668/psycharchives.327

Neuberger, O., & Allerbeck, M. (2014). *Arbeitszufriedenheit [Work satisfaction]*. ZIS. https://doi.org/10.6102/zis2

Schupp, J., & Gerlitz, J. Y. (2014). *Big Five Inventory-SOEP (BFI-S)*. Zusammenstellung sozialwissenschaftlicher Items und Skalen (ZIS). https://doi.org/10.6102/zis54

Westermann, R., Heise, E., & Spies, K. (2018). *FB-SZ-K - Kurzfragebogen zur Erfassung der Studienzufriedenheit [short scales to assess study satisfaction]*. ZPID (Leibniz Institute for Psychology) – Open Test Archive. https://www.psycharchives.org/handle/20.500.12034/562.3

Table S4. Bonferroni–Holm correlation adjustment procedure for extraversion and conscientiousness.

uncorrected *p*-value	construct 1	construct 2	*p*-value multiplication	corrected *p*-value
4.62043E-08	conscientiousness	positive affect	14	0.000
4.88154E-07	conscientiousness	satisfaction home study content	13	0.000
0.000203527	conscientiousness	grades	12	0.002
0.000225608	conscientiousness	general study satisfaction	11	0.002
0.001961042	conscientiousness	positive mood	10	0.020
0.006399737	conscientiousness	satisfaction university organization	9	0.058
0.026490095	conscientiousness	satisfaction home office	8	0.212
0.140746086	extraversion	satisfaction home office	7	0.985
0.346060616	extraversion	grades	6	2.076
0.349256623	extraversion	satisfaction home study content	5	1.746
0.737285501	extraversion	satisfaction university organization	4	2.949
0.821385604	extraversion	general study satisfaction	3	2.464
0.855842977	extraversion	negative mood	2	1.712

0.999423229	extraversion	negative affect	1	0.999

Table S5: Explorative correlation analysis without alpha adjustment.

		conscientiousness	extraversion	agreeableness	openness	neuroticism	positive affect	negative affect	ASTS sadness	ASTS hopelessness	ASTS fatigue	ASTS anger	negative mood	positive mood	general study satisfaction
**extraversion**	Pearson's r	0.156	—												
	df	285	—												
	p-value	0.008	—												
	Spearman's rho	0.152	—												
	df	285	—												
	p-value	0.01	—												
	N	287	—												
**agreeableness**	Pearson's r	0.153	0.089	—											
	df	285	285	—											
	p-value	0.01	0.133	—											
	Spearman's rho	0.141	0.067	—											
	df	285	285	—											
	p-value	0.017	0.255	—											
	N	287	287	—											
**openness**	Pearson's r	0.085	0.172	0.143	—										
	df	285	285	285	—										
	p-value	0.153	0.003	0.015	—										
	Spearman's rho	0.049	0.17	0.096	—										
	df	285	285	285	—										
	p-value	0.405	0.004	0.104	—										
	N	287	287	287	—										
**neuroticism**	Pearson's r	-0.103	-0.299	-0.071	0.179	—									
	df	285	285	285	285	—									
	p-value	0.081	< .001	0.229	0.002	—									
	Spearman's rho	-0.121	-0.301	-0.083	0.16	—									
	df	285	285	285	285	—									
	p-value	0.041	< .001	0.161	0.006	—									
	N	287	287	287	287	—									
**positive affect**	Pearson's r	0.309	0.205	0.138	0.093	-0.317	—								
	df	285	285	285	285	285	—								
	p-value	< .001	< .001	0.02	0.114	< .001	—								
	Spearman's rho	0.306	0.194	0.126	0.097	-0.337	—								
	df	285	285	285	285	285	—								
	p-value	< .001	< .001	0.033	0.1	< .001	—								
	N	287	287	287	287	287	—								
**negative affect**	Pearson's r	-0.175	-0.191	-0.154	0.136	0.522	-0.42	—							
	df	285	285	285	285	285	285	—							

	p-value	0.003	0.001	0.009	0.021	< .001	< .001	—					
	Spearman's rho	-0.165	-0.195	-0.14	0.128	0.522	-0.411	—					
	df	285	285	285	285	285	285	—					
	p-value	0.005	< .001	0.017	0.03	< .001	< .001	—					
	N	287	287	287	287	287	287	—					
**ASTS sadness**	Pearson's r	-0.183	-0.071	-0.043	0.154	0.406	-0.536	0.628	—				
	df	285	285	285	285	285	285	285	—				
	p-value	0.002	0.232	0.464	0.009	< .001	< .001	< .001	—				
	Spearman's rho	-0.163	-0.057	-0.03	0.156	0.411	-0.527	0.624	—				
	df	285	285	285	285	285	285	285	—				
	p-value	0.006	0.339	0.617	0.008	< .001	< .001	< .001	—				
	N	287	287	287	287	287	287	287	—				
**ASTS hopelessness**	Pearson's r	-0.173	-0.074	-0.036	0.137	0.367	-0.492	0.622	0.777	—			
	df	285	285	285	285	285	285	285	285	—			
	p-value	0.003	0.213	0.549	0.02	< .001	< .001	< .001	< .001	—			
	Spearman's rho	-0.174	-0.081	-0.022	0.133	0.366	-0.49	0.603	0.775	—			
	df	285	285	285	285	285	285	285	285	—			
	p-value	0.003	0.169	0.711	0.024	< .001	< .001	< .001	< .001	—			
	N	287	287	287	287	287	287	287	287	—			
**ASTS fatigue**	Pearson's r	-0.121	-0.041	-0.048	0.144	0.364	-0.484	0.587	0.725	0.724	—		
	df	285	285	285	285	285	285	285	285	285	—		
	p-value	0.04	0.486	0.414	0.014	< .001	< .001	< .001	< .001	< .001	—		
	Spearman's rho	-0.117	-0.057	-0.028	0.15	0.366	-0.484	0.597	0.708	0.724	—		
	df	285	285	285	285	285	285	285	285	285	—		
	p-value	0.047	0.335	0.632	0.011	< .001	< .001	< .001	< .001	< .001	—		
	N	287	287	287	287	287	287	287	287	287	—		
**ASTS anger**	Pearson's r	-0.043	0.073	-0.234	0.065	0.167	-0.311	0.459	0.576	0.593	0.515	—	
	df	285	285	285	285	285	285	285	285	285	285	—	
	p-value	0.467	0.218	< .001	0.274	0.005	< .001	< .001	< .001	< .001	< .001	—	
	Spearman's rho	-0.053	0.064	-0.202	0.088	0.171	-0.315	0.458	0.571	0.594	0.524	—	
	df	285	285	285	285	285	285	285	285	285	285	—	
	p-value	0.371	0.284	< .001	0.136	0.004	< .001	< .001	< .001	< .001	< .001	—	
	N	287	287	287	287	287	287	287	287	287	287	—	
**negative mood**	Pearson's r	-0.163	-0.033	-0.111	0.124	0.378	-0.565	0.648	0.901	0.887	0.844	0.773	—
	df	285	285	285	285	285	285	285	285	285	285	285	—
	p-value	0.006	0.576	0.06	0.035	< .001	< .001	< .001	< .001	< .001	< .001	< .001	—

	Spearman's rho	-0.162	-0.038	-0.086	0.13	0.379	-0.555	0.641	0.89	0.89	0.833	0.769	—		
	df	285	285	285	285	285	285	285	285	285	285	285	—		
	p-value	0.006	0.527	0.147	0.027	< .001	< .001	< .001	< .001	< .001	< .001	< .001	—		
	N	287	287	287	287	287	287	287	287	287	287	287	—		
**positive mood**	Pearson's r	0.17	0.024	0.11	0.005	-0.274	0.579	-0.387	-0.679	-0.585	-0.582	-0.527	-0.782	—	
	df	285	285	285	285	285	285	285	285	285	285	285	285	—	
	p-value	0.004	0.684	0.063	0.928	< .001	< .001	< .001	< .001	< .001	< .001	< .001	< .001	—	
	Spearman's rho	0.171	0.014	0.09	-0.021	-0.301	0.576	-0.384	-0.659	-0.579	-0.552	-0.498	-0.757	—	
	df	285	285	285	285	285	285	285	285	285	285	285	285	—	
	p-value	0.004	0.808	0.127	0.723	< .001	< .001	< .001	< .001	< .001	< .001	< .001	< .001	—	
	N	287	287	287	287	287	287	287	287	287	287	287	287	—	
**general study satisfaction**	Pearson's r	0.206	0.055	0.123	-0.126	-0.388	0.478	-0.513	-0.594	-0.618	-0.605	-0.475	-0.668	0.504	—
	df	285	285	285	285	285	285	285	285	285	285	285	285	285	—
	p-value	< .001	0.357	0.038	0.033	< .001	< .001	< .001	< .001	< .001	< .001	< .001	< .001	< .001	—
	Spearman's rho	0.193	0.063	0.07	-0.134	-0.385	0.473	-0.516	-0.584	-0.615	-0.607	-0.451	-0.642	0.48	—
	df	285	285	285	285	285	285	285	285	285	285	285	285	285	—
	p-value	0.001	0.286	0.239	0.023	< .001	< .001	< .001	< .001	< .001	< .001	< .001	< .001	< .001	—
	N	287	287	287	287	287	287	287	287	287	287	287	287	287	—
**grades total**	Pearson's r	-0.228	0.025	-0.025	-0.011	0.011	-0.11	0.05	0.027	0.142	-0.004	0.013	0.038	0.047	-0.154
	df	258	258	258	258	258	258	258	258	258	258	258	258	258	258
	p-value	< .001	0.692	0.684	0.861	0.861	0.078	0.42	0.67	0.022	0.949	0.836	0.545	0.448	0.013
	Spearman's rho	-0.274	0.041	-0.016	0.025	0.012	-0.133	0.048	0.059	0.165	-0.001	0.025	0.064	0.008	-0.214
	df	258	258	258	258	258	258	258	258	258	258	258	258	258	258
	p-value	< .001	0.508	0.793	0.686	0.847	0.033	0.439	0.347	0.008	0.981	0.684	0.305	0.893	< .001
	N	260	260	260	260	260	260	260	260	260	260	260	260	260	260

Note. * p < .05, ** p < .01, *** p < .001
